# Availability and effectiveness of decision aids for supporting shared decision making in patients with advanced colorectal and lung cancer: Results from a systematic review

**DOI:** 10.1111/ecc.13079

**Published:** 2019-05-08

**Authors:** Inge Spronk, Maartje C. Meijers, Marianne J. Heins, Anneke L. Francke, Glyn Elwyn, Anne van Lindert, Sandra van Dulmen, Liesbeth M. van Vliet

**Affiliations:** ^1^ Nivel (Netherlands Institute for Health Services Research) Utrecht the Netherlands; ^2^ Department of Public Health, Erasmus MC University Medical Center Rotterdam Rotterdam the Netherlands; ^3^ Health, Medical and Neuropsychology Unit, Institute of Psychology Leiden University Leiden the Netherlands; ^4^ Amsterdam Public Health Institute VU University Medical Centre Amsterdam the Netherlands; ^5^ The Dartmouth Institute for Health Policy and Clinical Practice Dartmouth Massachusetts; ^6^ University Medical Center Utrecht Utrecht the Netherlands; ^7^ Department of Primary and Community Care, Radboud Institute for Health Sciences Radboud University Medical Center Nijmegen the Netherlands; ^8^ Faculty of Health and Social Sciences University of South‐Eastern Norway Drammen Norway

**Keywords:** advanced colorectal cancer, advanced lung cancer, decision aid, shared decision making

## Abstract

**Introduction:**

Shared decision making is not always commonplace in advanced colorectal or lung cancer care. Decision aids (DAs) might be helpful. This review aimed (a) to provide an overview of DAs for patients with advanced colorectal or lung cancer and assess their availability; and (b) to assess their effectiveness if possible.

**Methods:**

A systematic literature search (PubMed/EMBASE/PsycINFO/CINAHL) and Internet and expert searches were carried out to identify relevant DAs. Data from the DAs included were extracted and the quality of studies, evidence (Grading of Recommendations Assessment, Development and Evaluation) and effectiveness (International Patient Decision Aid Standards) of DAs were determined.

**Results:**

Ten of the 12 DAs included (four colorectal cancer, four lung cancer and four generic) are still available. Most (9/12) were applicable throughout the disease pathway and usable for all decisions, or to the decision for supportive care with/without anti‐cancer therapy. Seven studies tested effectiveness. Effects on patient outcomes varied, but were generally weakly positive (e.g., DAs improved patient satisfaction) with low evidence. Study quality was fair to good.

**Conclusion:**

There is a lack of readily available DAs that have been demonstrated to be effective in advanced colorectal or lung cancer. Rigorous testing of the effects of currently available and future DAs, to improve patient outcomes, is urgently needed.

## 1 INTRODUCTION

Colorectal and lung cancer are common types of cancer (new worldwide cases in 2018: 1.8 and 2.1 million respectively) with—depending on the tumour stage—unfavourable prognoses (International Agency for Research on Cancer, 2018a, 2018b). Patients for whom curative treatment options are not or are no longer possible often face difficult and preference‐sensitive treatment and/or care decisions affecting life expectancy and quality of life. Shared decision making (SDM) can help make these decisions, including decisions to forego active cancer treatment (Legare, Ratte, Gravel, & Graham, 2008).

Shared decision making is an approach in which patients and clinicians discuss the best available evidence when facing decisions, while patients are assisted in expressing their preferences and becoming actively involved in decision making (Elwyn et al., 2012, 2010; Longtin et al., 2010). SDM is an important element of high‐quality cancer care, with essential elements including acknowledging patients' informed values (Stacey, Samant, & Bennett, 2008) and understanding patients’ care goals (Bernacki & Block, 2014; Kane, Halpern, Squiers, Treiman, & McCormack, 2014). It is appreciated by many patients (Degner & Sloan, 1992; Keating, Guadagnoli, Landrum, Borbas, & Weeks, 2002) and has been associated with positive patient outcomes, such as increased knowledge about the available options, better perceived quality of care and improved quality of life (Kashaf & McGill, 2015; Kehl et al., 2015; Stacey et al., 2017). In advanced cancer, decision making is particularly influenced by personal values and cannot be ruled by evidence‐based medicine alone (Bélanger, Rodríguez, & Groleau, 2011; Reyna, Nelson, Han, & Pignone, 2015). However, despite political and clinical support for the SDM approach, uptake in clinical practice has been slow (Brom et al., 2017; Coulter, Edwards, Elwyn,& Thomson,^2011^).

For enhancing the process of actively involving patients in SDM, using decision aids (DAs) might be helpful (van Weert et al., 2016). DAs are tools that help patients to come to the best decision by showing the available options (treatment and care options), clarifying personal values and providing information about the available options and their outcomes (Waitzkin, 1985). DAs are available in various forms such as patient letters, video or audiotapes, leaflets, computer programs or interactive media (Stacey et al., 2014). In essence, they encourage patients to think about their preferences for future treatment and care. Exploring options using DAs helps cancer patients form more stable preferences (Pieterse et al., 2011), improves their knowledge and awareness of treatment options (Austin, Mohottige, Sudore, Smith, & Hanson, 2015), enhances patient involvement in decision making (Kashaf & McGill, 2015; Kunneman et al., 2015; Stacey et al., 2008) and improves quality‐of‐life outcomes (Bernacki & Block, 2014; Kashaf & McGill, 2015).

Decision aids might be promising for advanced colorectal and lung patients who face difficult and preference‐sensitive treatment decisions, a group that is growing (Cronin et al., 2018). There are no overviews of which DAs are available for these patients and whether these DAs affect patient outcomes. This review therefore aims (a) to provide an overview of DAs for patients with advanced colorectal or lung cancer and assess their availability; and (b) to assess their effectiveness if possible.

## 2 METHODS

This systematic review was conducted and reported in line with the PRISMA Statement (Moher, Liberati, Tetzlaff, & Altman, 2009) and registered in PROSPERO (ID = CRD42018094453). Two strategies were used to identify DAs for patients with advanced colorectal or lung cancer: (a) a systematic literature search; (b) an Internet search and expert consultation.

### 2.1 Search strategy

#### 2.1.1 Systematic literature search

PubMed, EMBASE, PsycINFO and CINAHL were searched to identify relevant articles published between January 2006 and March 2018 (comparable to what was done by Spronk, Burgers, Schellevis van Vliet, and Korevaar (2018)). We used this timeframe because we were looking for DAs that are still relevant. Older DAs that are still relevant would have been found through the Internet search and when consulting the experts, or through manual searching of reference lists. The search strategy (Appendix 1) was developed in collaboration with an experienced librarian and checked by an expert in the field (Glyn Elwyn). A manual search of reference lists of the articles included was conducted to identify additional relevant articles.

#### 2.1.2 Internet search and consultation of experts

The Internet search and expert consultation complemented the systematic literature search, as we hypothesised that not all the DAs might have been published in peer‐reviewed journals (or not yet). Internet searches covering the topics “advanced colorectal or lung cancer” and “decision making” were carried out in Google (Appendix 2) in 2018 on the 21st of March and the first four pages of results were screened (comparable to what was done by Van Vliet, Harding, Bausewein, Payne, and Higginson (2015)). In addition, websites including overviews of DAs (http://www.med-decs.org/, https://decisionaid.ohri.ca/) were screened on the same day. Lastly, experts were contacted by e‐mail to identify available DAs for patients with advanced colorectal or lung cancer. Experts were international SDM experts (*n* = 6, from Australia, Canada, Norway, the United Kingdom and the USA) and Dutch SDM, colorectal cancer and lung cancer experts (*n* = 13). They were identified via core articles or through the research team's own network.

### 2.2 Inclusion criteria

#### 2.2.1 Systematic literature search

We defined our research question according to the PICO criteria:

Original empirical published studies, written in any language, were included if they focused on:

Participants: adult (>18 years) patients with advanced colorectal or lung cancer (i.e., patients for whom curative treatment options are no longer possible).
Intervention: development and/or evaluation of a DA that focused on (a) providing information about current options; (b) current decision making processes; or (c) helping patients by eliciting preferences for current treatment options.
Comparison: for our second research question, that is the effectiveness of DAs, studies were included if they included a comparison (e.g., standard care) and also when there was no comparison group (e.g., pre‐test, post‐test design).
Outcomes: for our second research question, that is the effectiveness of DAs, any patient‐reported outcome (e.g., satisfaction with decision) and/or health outcomes (e.g., general health).

#### 2.2.2 Internet search and consultation of experts

The same patient and intervention inclusion criteria were applied as for the systematic literature search. However, we anticipated that the comparison and outcome inclusion criteria would not apply.

### 2.3 Study selection and data extraction

#### 2.3.1 Systematic literature search

One researcher (IS) performed the search and removed duplicates. Two researchers (IS and LvV) independently screened 15% of the records based on title and abstract. The overlap was 100%, so the additional records were screened by a single researcher (IS). In the case of any doubt, the record was included and screened by two authors independently during full‐text screening. Full‐text screening and extraction of data was done independently by two researchers (IS and MH/MM). The information extracted included study characteristics (first author, year of publication, study size, study design, patient characteristics, outcome measures [if present]), characteristics of the DA (name, description, target population, country, options on which the DA focuses), and patient‐reported outcomes and health outcomes (if present). In the case where a DA was not included in the article, or not found on the Internet, the authors/developers were contacted about its status and asked to send the researchers a copy of the DA. Disagreements arising from decisions around article inclusion or the extraction of data were discussed with a third researcher (LvV). When consensus was not reached with the third author, the research team was involved and the issue was discussed until consensus was reached.

#### 2.3.2 Internet search and consultation of experts

The Internet search was carried out by one researcher (IS). Potentially relevant DAs were selected and independently screened by two researchers (IS and MH/LvV). DAs provided by the experts were handled in the same way. The data extraction followed the same steps as used in the systematic literature search.

### 2.4 Quality assessment

#### 2.4.1 Quality of included studies

As the included studies used different designs, their quality was assessed with the quality assessment tool of Hawker, Payne, Kerr, Hardey, and Powell (2002). This tool includes nine domains: abstract and title; introduction and aims; method and data; sampling; data analysis; ethics and bias; results; transferability and implications/usefulness. Following Hawker et al. (2002), each domain was assessed for each study, with scores ranging from 1 (“very poor”) to 4 (“good”). The total score ranges between 9 and 36 points. Scores up to 18 points are rated as “poor quality”; scores between 19 and 27 as “fair quality”; scores above 27 as “good quality” (Appendix 3). Each study was independently assessed by two researchers (MH and AF/SvD). A threshold of five points was used; if the overall quality scores differed more than five points, the average was calculated (comparable to the way it was done by Voss et al. (2017).

#### 2.4.2 Level of evidence DAs included

To assess the level of evidence of the DAs, the Grading of Recommendations Assessment, Development and Evaluation (GRADE) methodology was used (Guyatt et al., 2008). GRADE classifies evidence into four quality levels (high, moderate, low and very low). Studies were classified based on their design. Randomised control trials (RCTs) get a high‐quality initial grade and observational studies a low‐quality initial grade. These initial grades can be upgraded or downgraded after assessment of their strengths and weaknesses. Risk of bias, indirectness of evidence, inconsistency of results, imprecision in the results and publication bias are criteria for downgrading, whereas a large magnitude of effect, dose–response and opposing residual confounding or bias are criteria for upgrading. Based on the upgrading and downgrading criteria, the final evidence grade was independently determined by two researchers (IS and MH). Disagreements were resolved by discussion with a third researcher (LvV).

#### 2.4.3 Effectiveness of the DAs included

To evaluate the effectiveness of the DAs, “part III Effectiveness” of the International Patient Decision Aid Standards (IPDAS) criteria for judging the quality of patient DAs was used (Elwyn et al., 2006). This part consists of seven items. These items include assessment of whether the DA helps patients (a) to recognise that a decision needs to be made; (b) to know the options and their features; (c) to understand that values affect the decision; (d) to be clear about which features of the options matter most; (e) to discuss values with their practitioner, 6) to become involved in the patients’ preferred way; and (g) to improve the match between the chosen option and the features that matter most to the properly informed patient. If an item is fulfilled, a score of 1 is given. Total scores could range between 0 and 7 points. Two researchers (IS and MM) independently scored the IPDAS. Disagreements were resolved by discussion with a third researcher (LvV).

## 3 RESULTS

**Figure 1:**
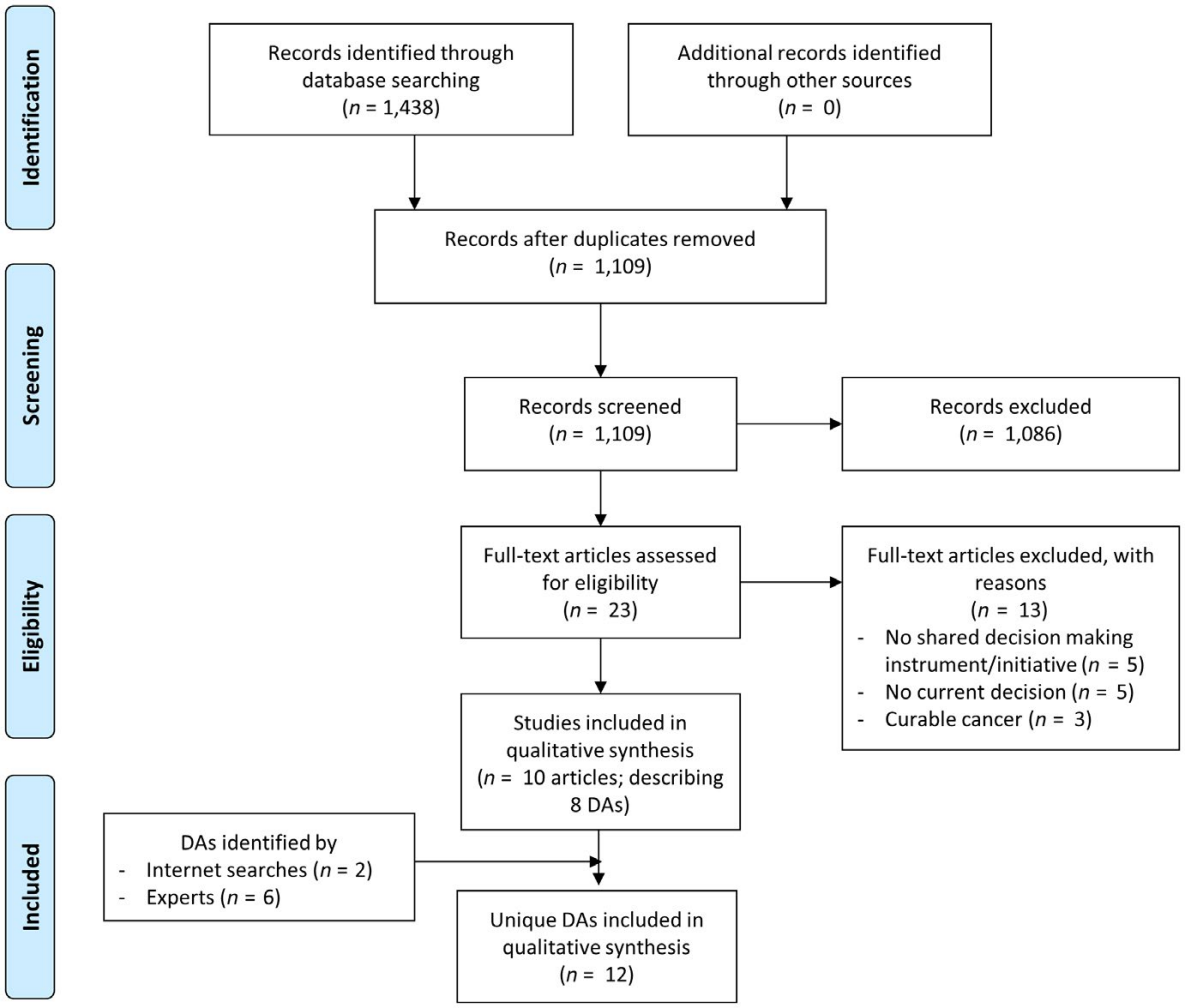
Flow chart of the inclusion of decision aids (DAs)

The initial literature search resulted in 1,438 potentially relevant articles. After removal of duplicates and elimination of articles based on title abstract screening, the full texts of 23 articles were screened. Thirteen of these did not meet our inclusion criteria, resulting in the inclusion of 10 articles describing eight unique DAs (Figure 1). The Internet search revealed two relevant DAs and the experts suggested six DAs. Four of these eight DAs had not been identified by the systematic search and were therefore added (Figure 1).

Table 1 gives an overview of the main characteristics of all DAs (*n* = 12) that were included. Four DAs were specifically designed for patients with advanced colorectal cancer (Enzinger et al., 2017; Leighl et al., 2011; Maag Lever Darm Stichting (Dutch digestive disease foundation), 2016; Oostendorp et al., 2017), four were designed for advanced lung cancer patients (DuBenske, Gustafson, Shaw, & Cleary, 2010; MAASTRO clinic, 2018; Steendam, Schaffelaars, Belderbos, & Pruyn, 2016; Tang et al., 2008) and the other four were not disease‐specific (Henselmans et al., 2018; Meropol et al., 2013; Shirai et al., 2012; Smith et al., 2011). Five had been developed in the Netherlands, four in the USA, one in Singapore, one in Japan, and one was developed by collaborating researchers from both Australia and Canada. All the DAs had been developed to be used by patients before the consultation; none were designed to be used during the consultation. Only one DA (Meropol et al., 2013) engaged the clinician, who received a summary report of the patient's responses that could then be used during the consultation.

**Table 1:** Overview of DAs for shared decision making in advanced colorectal and lung cancer

Name decision aid/short description	First author/developer	Year developed	Country	Source	Description of tool
Colorectal cancer					
Decision aid for second‐line chemotherapy	Oostendorp (Oostendorp et al., 2017)	2017	Netherlands	E, I, S	A DA (booklet) describing the adverse events, response of the cancer and survival of supportive care with or without second‐line palliative chemotherapy
Decision aid for first‐line chemotherapy	Leighl (Leighl et al., 2011)	2011	Australia and Canada	E, S	A DA (booklet with accompanying audiotape) presenting options of supportive care, with or without chemotherapy. Potential benefits and side effects of different chemotherapy regimens, and evidence‐based prognostic estimates are described, and a value clarification exercise is included
A prototype video and companion booklet supporting informed consent	Enziger (Enzinger et al., 2017)	2017	USA	S	A prototype (regimen‐specific chemotherapy informed consent) video and companion booklet (explaining guideline‐recommended treatment options for metastatic colorectal cancer) supporting informed consent for a common palliative chemotherapy regimen
MLDS decision aid	MLDS (Maag Lever Darm Stichting (Dutch digestive disease foundation), 2016)	2016	Netherlands	E, I	Website providing information (including videos) and an instrument for patient’s value clarification of which a summary is made to discuss with the physician.
Lung cancer					
Maastro decision aid	MAASTRO clinic (MAASTRO clinic, 2018)	2018	Netherlands	E	A DA (website) for lung cancer patients (stage), that describes characteristics, side effects and differences between surgery and radiotherapy, and assists patients to think about their preferences and values so they can discuss their preferences with their clinician and make an informed decision
Decision board	Tang (Tang et al., 2008)	2008	Singapore	S	A decision board outlining the various advantages and disadvantages of Fx schedules (17 Gy in two fractions vs. 39 Gy in 13 fractions), in the palliation of symptomatic unresectable lung cancer
Decision Aid for stage 4 lung cancer	Steendam (Steendam et al., 2016)	2016	Netherlands	E	A tool for patients with advanced lung cancer and their relatives, which includes an introductory letter, presentation of potential pros and cons of the treatment options (palliative chemo, immunotherapy, or experimental treatment or supportive care), most common side effects, and a personal DA for making difficult decisions
*Comprehensive Health Enhancement Support System (CHESS)* ^a^	*DuBenske (*DuBenske et al., 2010 *)*	*2010*	*USA*	*S*	*A Web‐based interactive health communication system (IHCS)—(CHESS)—for patients with advanced lung cancer and their family caregivers, which provides information, communication, and coaching resources as well as a symptom tracking system that reports health status to the clinical team*
Not colorectal or lung cancer specific					
Question prompt sheet (QPS)	Shirai (Shirai et al., 2012)	2012	Japan	S	A question prompt sheet (63 questions) to facilitate the involvement (by preparing questions prior to consultation) of advanced cancer patients during consultations
Consultation guide CHOICE	Henselmans (Henselmans et al., 2018)	2016	Netherlands	E	A booklet with sample questions to facilitate shared decision making and an instrument for value clarification
Decision aid for first‐, second‐, third‐ and fourth‐line chemotherapy	Smith (Smith et al., 2011)	2013	USA	S	State‐of‐the‐art tables with information for patients with advanced breast, lung, colon and hormone‐refractory prostate cancers facing first‐, second‐, third‐ and fourth‐line chemotherapy.
*CONNECT*	*Meropol (*Meropol et al., 2013 *)*	*2013*	*USA*	*S*	*A communication aid that assesses patient values (quality of life), goals, and communication preferences, and includes communication skills training, plus a pre‐consultation summary report to the physician*

Note

Tools that are no longer available are printed in italics.

Source: S = systematic search, E = experts, I = Internet search, DA = decision aid.

### 3.1 Colorectal cancer DAs

All four of the DAs for patients with advanced colorectal cancer are still available. Two DAs included booklets presenting options for supportive care with or without first‐line (Leighl et al., 2011) or second‐line (Oostendorp et al., 2017) chemotherapy (Table 1). The booklet of Leighl et al. was accompanied by an audiotape. The third DA, a booklet accompanied by a video, included the informed consent process regarding palliative chemotherapy (Enzinger et al., 2017), and the fourth DA (Decision aid MLDS) (Maag Lever Darm Stichting (Dutch digestive disease foundation), 2016) is a website (including videos) about patients’ value clarification in the palliative phase of their disease.

The effectiveness of two DAs focusing on supportive care with or without first‐ or second‐line chemotherapy was tested by comparing them in RCTs against standard care (Leighl et al., 2011; Oostendorp et al., 2017) (Table 2). Patients receiving the DA on first‐line chemotherapy (Leighl et al., 2011) demonstrated higher overall understanding of the prognoses but satisfaction was similar to the control group (quality: good, GRADE: moderate, IPDAS: 6/7). Patients receiving the DA on second‐line chemotherapy (Oostendorp et al., 2017) were no less anxious and did not perceive better well‐being compared to the control group (quality: good, GRADE: moderate, IPDAS: 3/7). A third DA (Enzinger et al., 2017) was developed for advanced colorectal cancer patients. It was, however, not evaluated in this patient group.

**Table 2:** Characteristics of evaluated decision aids (DAs), including the quality, Grading of Recommendations Assessment, Development and Evaluation (GRADE) and International Patient Decision Aid Standards (IPDAS) scores

Name of decision aid/short description	First author (year)	Study population *n* (sex), age	Design	Decision aid outcome measures	Outcome	Quality^a^	GRADE	IPDAS
Colorectal cancer								
Decision aid for second‐line chemotherapy	Oostendorp (2017) (Oostendorp et al., 2017)	Patients with metastatic colorectal or breast cancer, *n* = 128 (F: 63%), mean age: 61 years	RCT	Primary: (well‐being) anxiety Secondary: (well‐being) depression, general health, cancer worries, health‐related quality of life Additional: coping styles, amount of information received, satisfaction with the quality of information, subjective knowledge, treatment preference, decision satisfaction and uncertainty, decision control and treatment attitudes	No statistically significant differences in anxiety No statistically significant differences in depression, general health, cancer worries, health‐related quality of life Use of the DA was associated with stronger treatment preferences (*p* = 0.030) and increased subjective knowledge (*p* = 0.022) No statistically significant differences in coping styles, amount of information received, satisfaction with quality of information, decision satisfaction and uncertainty, decision control and treatment attitudes	Good	Moderate	3
Decision aid for first‐line chemotherapy	Leighl (2011) (Leighl et al., 2011)	Patients with advanced colorectal cancer, *n* = 208 (F: 46%), median age: 61 years	RCT	Primary: patient understanding of prognostic and treatment information and satisfaction with decision making Additional: decisional conflict, anxiety, quality of life, treatment decision made, patient achievement of decision involvement preferences	Patients receiving the DA demonstrated a greater increase in understanding of prognosis and the palliative goals of treatment, with higher overall understanding (*p* = 0.001) No statistically significant differences in satisfaction with decision making No statistically significant differences in decisional conflict, quality of life, treatment decision made and preferences for decision involvement Patient anxiety (was low to moderate at all time points) did not differ between study arms	Good	Moderate	6
Lung cancer								
Decision board	Tang (2008) (Tang et al., 2008)	Unresectable lung cancer patients, after diagnosis, *n* = 92 (F: 24%), median age: 68 years	Uncontrolled, observational study	Primary: patient's preferred Fractionation schedule (17 Gy in two fractions vs. 39 Gy in 13 fractions), Secondary: patients’ reasons and their level of satisfaction with being involved in the decision making process.	Fifty‐one patients indicated a preference for 39 Gy in 13 fractions and 41 chose 17 Gy in two fractions after going through the decision board process Longer Fx was chosen because of longer survival (90%) and better local control (12%). Shorter Fx was chosen for shorter overall treatment duration (80%), cost (61%) and better symptom control (20%) All patients (100%) were satisfied with being involved in the decision making process	Fair	Very low	5
*CHESS*	*DuBenske (2010) (*DuBenske et al., 2010 *)*	*Non‐small cell lung cancer, after diagnosis, n = 285 (F: 50%), median age: 62 years*	*RCT*	*Primary: patient symptom distress measured by caregivers*	*Caregivers in the CHESS arm consistently reported lower patient physical symptom distress (at 4 months [p = 0.031; Cohen d = 0.42] and at 6 months ]p = 0.004; d = 0.61])* *Marginally significant differences at 2 months (p = 0.051; d = 0.39) and at 8 months (p = 0.061; d = 0.43)*	*Fair*	*Moderate*	*6*
Not lung or colorectal cancer specific								
Question prompt sheet (QPS)	Shirai (2012) (Shirai et al., 2012)	Advanced cancer patients (lung, gastric, colorectal, oesophageal, *n* = 63 (F: 34%), median age 64 years	RCT	Primary: patient rating of the usefulness of the material(s) Secondary: satisfaction with the consultation, number of questions overall and frequency of questions	Patients gave a greater usefulness score for the materials (to ask questions [*p* = 0.033]; to understand the treatment plan [*p* = 0.051]; willingness to use material in future [*p* = 0.006]) No statistically significant differences in satisfaction with the consultation No statistically significant differences in number of total questions and frequency of type of questions	Good	Moderate	3
Decision aid for first‐, second‐, third‐ and fourth‐line chemotherapy	Smith (2011) (Smith et al., 2011)	Patients with metastatic breast, colorectal, lung, or prostate cancer, *n* = 27 (F: 56%), mean age: 63 years	Pilot pre‐test, post‐test study	Primary: Number of patients who opt for full disclosure once they viewed the DA Secondary: the amount of information patients have about cure, response rates, and symptom control; the impact of truthful information on hope, whether the information was deemed helpful to the patient; and whether the patient wants to share the information with a physician	96% (26/27) of the patients chose to complete the DA The proportion of patients who thought that advanced cancer could be cured reduced from 52% to 32% (*p* = 0.15) Patients became only slightly less overoptimistic about response rate and symptom control (not significant) No distress was noted and hope did not change 93% found the information helpful 74% wanted to share the information with their family and physician	Fair	Very low	1
*CONNECT*	*Meropol (2013) (*Meropol et al., 2013 *)*	*Metastatic cancer patients,* *n = 629, (F: 48%),* *mean age: 59 years*	*RCT with 3 arms* *(1 control, 2 intervention)* b	*Treatment outcome expectations, decisional conflict, patient satisfaction with the content and format of the communication, and satisfaction with the survey and/or communication skills training* c	*Patients were less likely to believe that they would experience severe side effects with standard or experimental therapy (p < 0.05)* *Treatment decisions were easier to reach (p = 0.003)* *Patients were more satisfied with decisions (p < 0.001)* Patients were more satisfied with the physician communication format (p = 0.026) Patients were more satisfied with the discussion regarding support services (p = 0.029) and quality of life concerns (p = 0.042) No statistically significant differences in satisfaction regarding discussion of diagnosis/prognosis, treatment options, support/community services, and decisional conflict scores	*Good*	*Low*	*7*

Note

Tools that are no longer available are printed in italics.

Study population: *n* = sample size; *F* = female.

a Assessed with the quality assessment tool of Hawker et al. (2002).

b The final analysis was on two arms: (1) control group (2) CONNECT with physician summary & CONNECT without physician summary.

c Measures and outcomes described as in the article. Please note that the overlap is not complete.

### 3.2 Lung cancer DAs

Three of the four DAs identified for advanced lung cancer are still available. One DA consisted of a website that is still being developed and that describes characteristics, side effects and differences between surgery and radiotherapy; it assists patients in thinking about their preferences and values to let them make an informed decision (MAASTRO clinic, 2018). A second DA comprised a decision board (Tang et al., 2008) about the advantages and disadvantages of various radiation schedules. Lastly, the third DA consisted of a booklet for stage 4 lung cancer patients (Steendam et al., 2016) about the potential treatment options (including chemotherapy, immunotherapy and experimental studies) versus supportive care without anti‐cancer therapy. The DA of DuBenske et al. (2010) (CHESS) is no longer available. This DA comprised an interactive communication system to bridge the communication gaps that occur between patients, families and clinicians in cancer care in order to enhance SDM.

The effectiveness of two out of the four DAs was tested (DuBenske et al., 2010; Tang et al., 2008), although they differed substantially in terms of study design, content and outcome measures. The CHESS DA (DuBenske et al., 2010) was tested in an RCT and compared against a control group that received standard care and had access to the Internet. Using CHESS resulted in significantly lower distress in patients (*p* = 0.031; quality: fair, GRADE: moderate, IPDAS: 6/7). The decision board (Tang et al., 2008) was tested in an observational study with a suboptimal design that had no control group and in which the description of the outcome measures was deficient. Evaluation showed that all patients (100%) were satisfied with being involved in the decision making process (quality: fair, GRADE: very low, IPDAS: 5/7).

### 3.3 Generic DAs used by colorectal and lung cancer patients

The four other DAs were generic for all cancer types but were used in advanced colorectal and/or lung cancer patients. Three of these are still available. The first DA is a communication aid (Shirai et al., 2012) that includes a question prompt sheet that can be used by patients during a consultation. The other two DAs consist of a booklet with either sample questions accompanied by an instrument about value clarification (currently being evaluated) (Henselmans et al., 2018) or a booklet with tables including information about first‐, second‐, third‐ and fourth‐line chemotherapy (Smith et al., 2011). The CONNECT DA (Meropol et al., 2013) is not available anymore. This DA was a communication aid for patients and assessed their values, goals and communication preferences, alongside communication skills training. This was the only DA identified that engaged the healthcare provider by providing them with a summary report of the patient's responses.

Three of the generic DAs were evaluated. Two were tested in an RCT comparing them against standard care (Meropol et al., 2013; Shirai et al., 2012), and one was tested in a pilot study without a control group but with a pre‐test/post‐test design (Smith et al., 2011). The DA of Meropol et al. significantly increased patient satisfaction, while making it easier to reach decisions compared to standard care (quality: good, GRADE: low, IPDAS 7/7). Patients rated the materials of the DA of Shirai et al. (2012) as useful, but the DA did not lead to statistically significant differences in the overall numbers of questions posed and the frequency of questions compared to standard care (quality: good, GRADE: moderate, IPDAS: 3/7). The information tables (Smith et al., 2011) were felt to be helpful (74%). Patients were willing to complete the DA (96%) and share the information with their physician (93%), which might result in SDM. That being said, 31% of the patients thought that their cancer could be cured and 87% overestimated the positive effects of palliative chemotherapy (quality: fair, GRADE: very low, IPDAS: 1/7).

## 4 DISCUSSION

The aim of this systematic review was to provide an overview of DAs for patients with advanced colorectal or lung cancer and to assess their availability and effectiveness. This is a highly under‐researched area, despite patients facing multiple preference‐sensitive decisions affecting survival time and quality of life. Twelve DAs were identified (evenly distributed between colorectal, lung and generic cancer DAs), of which 10 are still available. Only seven of the DAs have been evaluated, and the effectiveness on patient outcomes was limited. Moreover, the quality of the DAs and the evidence was impaired (low to moderate) due to many forms of biases, limiting the certainty with which firm conclusions can be drawn about the DAs’ effectiveness.

Our systematic review first illustrates that there is a lack of readily available DAs for use in advanced colorectal and lung cancer care. This is in contrast to the earlier phases of the cancer pathway. In a systematic review, conducted in 2014, 55 available DAs—across various cancer types—were found (Trikalinos, Wieland, Adam, Zgodic, & Ntzani, 2014). Of the 10 available tools that were identified, some were still in the development or testing phase (Henselmans et al., 2018; MAASTRO clinic, 2018) and another was over a decade old and no update seems to have occurred (Tang et al., 2008). Whether or not the other tools were updated after publication remains unclear. This might be problematic, as guidelines change over time and more evidence about the recommended treatment of choice may become available. Moreover, two of the DAs that improved patient outcomes such as physical distress (DuBenske et al., 2010) and decision making/communication satisfaction (Meropol et al., 2013) were no longer available due to a lack of funding to keep the DAs available and up to date (personal communication). These results are in line with two related, recently published systematic reviews of DAs in advanced breast and other cancers (Spronk, Burgers, et al., 2018; Tapp & Blais, 2018), which also found few available, up‐to‐date DAs. For example, four out of the sixteen identified DAs for advanced cancer had not been updated in the last 15 years (Tapp & Blais, 2018). This seem to contrast with the push from many governments to endorse the use of DAs to improve clinical SDM and the quality of care provided (Australian Commission on Safety Quality in Health Care, 2015; Department of Health, 2010; Saskatchewan Health Quality Council, 2009; United States Federal Statute, 2010).

Before the clinical use of DAs can be widely recommended for patients with advanced colorectal and lung cancer, it is essential that they have demonstrated the ability to improve patient outcomes. Our systematic review provided little unequivocal evidence that this is the case in advanced colorectal and lung cancer patients. Some positive effects were found, for example on subjective knowledge (Oostendorp et al., 2017), prognostic understanding (Leighl et al., 2011), and satisfaction with communication and decision making (Meropol et al., 2013). Many of the outcomes studied, however, remained unaffected and the quality of the evidence was suboptimal, making it difficult to draw firm conclusions. These limitations hold for many DAs in advanced cancer, as similar conclusions were reached by the above‐mentioned systematic reviews (Spronk, Burgers, et al., 2018; Tapp & Blais, 2018).

While the aim of DAs is to improve patient outcomes, it is equally important to ascertain that their use is not harmful. We found that the DAs included did not increase patients’ psychological distress (e.g., anxiety (Leighl et al., 2011; Oostendorp et al., 2017)) or diminish patients’ hope (Smith et al., 2011). These findings illustrate that clinicians might not need to worry that using DAs will negatively affect their patients’ well‐being, but should also not be too optimistic that it improves their outcomes. These findings are in line with a recent updated Cochrane review of SDM initiatives, in which uncertain evidence from available DAs and related tools on patient outcomes was found. (Légaré et al., 2018). This underlines the need for more high‐quality studies in this quickly evolving research field to guide clinical practice and policy further.

Several recommendations can be made for optimising the development and evaluation of current and future DAs in advanced colorectal and lung cancer care. First, improvements of current DAs and development of future DAs should preferably be done in collaboration with national and international medical and physicians’ associations, which also take ownership and responsibility for keeping the DAs up to date. Using the best available evidence and guidelines (like IPDAS) to provide information for the development phase should also improve the quality of DAs (Durand et al., 2015; Elwyn et al., 2006; Joseph‐Williams et al., 2014). Second, it is essential to understand whether DAs improve SDM in clinical practice, and subsequently patient outcomes. Only few current studies assessed whether DAs actually improve SDM (Stacey et al., 2017). Previous studies showed that DAs used by patients before the consultation often lead to a better understanding of the options, but do not guarantee SDM (Hargraves & Montori, 2014; Stiggelbout et al., 2012). Focusing on the link between SDM and patient outcomes, SDM in colorectal or lung cancer (irrespective of patients’ preferences for SDM) improves the evaluated quality of received communication and provided care from the patient's perspective (Kehl et al., 2015). In other settings, tools (e.g., Option Grids) have been developed that can be used by the patient and clinician together during a clinical visit to ensure SDM and to improve patient outcomes (Breslin, Mullan, & Montori, 2008; Elwyn et al., 2013). Such tools might be useful for improving SDM and patient outcomes in advanced colorectal and lung cancer care. Third, according to an expert group of clinicians, researchers and patient representatives (Spronk, van Dulmen, Heins, & van Vliet, 2018), several preconditions at the level of the organisation (e.g., enough time (Legare et al., 2008), professional (e.g., a perceived added value of SDM), patient (e.g., insight into options) and patient–clinician interaction (continuous check of patient preferences) need to be met in order for SDM initiatives such as DAs to be successful (van Vliet et al., 2018). Fourth, patients and patient associations need to be involved from development through to implementation in order to ensure the DA is useful and understandable (Montori, Breslin, Maleska, & Weymiller, 2007).

### 4.1 Strengths and limitations

This review has strengths and limitations. A strength is the comprehensive overview, including all languages and the fact that a systematic literature search was conducted alongside an Internet and expert inventory. Four medical and social science databases were searched using a systematic search strategy that was developed in collaboration with an experienced librarian and checked by an expert in the field. A limitation is that only some of the DAs were evaluated and that we did not assess patients’ and clinicians’ views on the included DAs. In addition, the title/abstract screening of our systematic review was predominantly (85%) done by a single researcher, which could potentially have led to studies being missed. However, in the case of any doubt during the title/abstract screening, the record was included and screened by two authors independently during full‐text screening. Manual searches of the reference lists of articles included were conducted in order to identify potentially missed relevant studies. Limitations at the study level include the generally low quality of evidence of the DAs included, which was due to multiple sources of bias (e.g., study design, small sample sizes, high drop‐out rates, presentation of selective results). This may have skewed the results. Limitations at the outcome level include the various outcome measures across studies that impeded comparison of DAs at the outcome level. Finally, we primarily consulted Dutch experts, which may have caused bias in the identification of unpublished work.

## 5 CONCLUSION

To conclude, there is a shortage of readily available DAs with demonstrated positive effects on patient outcomes in advanced colorectal or lung cancer. Rigorous testing is needed of the effects of DAs that have not yet been tested in proper designs (possibly after updating), DAs that are currently under development, and DAs that may be developed in the future. Such initiatives are urgently needed in order to inform and shape the worldwide focus on using DAs and improving SDM in clinical care and to ensure patient outcomes are improved.

## CONFLICT OF INTEREST

No conflicts of interest to declare.

## APPENDIX 1

### SEARCH STRATEGY

Search in Pubmed (date: 16 March 2018)

**Table:**

Search strategy		Number of hits
Colorectal cancer		
#1	"colorectal cancer"[tiab]	
#2	colorectal neoplasms[mesh]	
#3	"colon cancer"[tiab]	
#4	"rectal cancer"[tiab]	
#5	"rectum cancer"[tiab]	
#6	"adenoma cancer"[tiab]	
Lung cancer		
#7	"lung cancer"[tiab]	
#8	"non‐small cell lung cancer"[tiab]	
#9	"non small cell lung cancer"[tiab]	
#10	"small cell lung cancer"[tiab]	
#11	lung neoplasms[mesh]	
#12	#1 OR #2 OR #3 OR #4 OR #5 OR #6 OR #7 OR #8 OR #9 OR # #10 OR #11	446,543
Advanced care		
#13	palliative care[mesh]	
#14	palliative[tiab]	
#15	Hospice Care[mesh]	
#16	hospice[tiab]	
#17	end‐of‐life[tiab]	
#18	terminal[tiab]	
#19	incurable[tiab]	
#20	Terminal Care[mesh]	
#21	"early palliative care"[tiab]	
#22	"serious illness"[tiab]	
#23	"advanced cancer"[tiab]	
#24	"metastatic cancer"[tiab]	
#25	metastasis[tiab]	
#26	Neoplasm Metastasis[MeSH Terms]	
#27	#13 OR #14 OR #15 OR #16 OR #17 OR #18 OR #19 OR #20 OR #21 OR #22 OR #23 OR #24 OR #25 OR #26	816,032
Decision making		
#28	"decision making"[tiab]	
#29	"decision support"[tiab]	
#30	"decision aid*"[tiab]	
#31	"choice behavior"[tiab]	
#32	"choice behaviour"[tiab]	
#33	(((((shared)[tiab] OR sharing)[tiab] OR informed[tiab]))) AND ((decision*[tiab]) OR choice*[tiab])	
#34	#28 OR #29 OR #30 OR #31 OR #32 OR #33	131,245
#35	#12 AND #27 AND #34	512
	limit #35 to (humans and yr="2006–2017")	397

Note

This initial search strategy was adapted to Cinahl, Medline and PsychInfo.

## APPENDIX 2

### INTERNET SEARCH

Internet search in Google (date: 21 March 2018).

**Table:**

Internet search		Number of hits
Search 1		
#1	Shared decision making	
#2	Lung cancer	
#3	Colorectal cancer	
	#1 AND (#2 OR #3)	26,100,000
Search 2		
#4	Decision aid	
#5	Lung cancer	
#6	Colorectal cancer	
	#4 AND (#5 OR #6)	8,870,000
Search 3		
#7	Decision support	
#8	Lung cancer	
#9	Colorectal cancer	
	#7 AND (#8 OR #9)	26,900,000
Search 4		
#10	Shared decision making	
#11	Decision aid	
#12	Decision support	
#13	Advanced cancer	
#14	Palliative cancer care	
	(#10 OR #11 OR #12) AND (#13 OR #14)	4,310,000

## APPENDIX 3

Quality appraisal scores

**Table:**

Author (Year)	Abstract and title	Introduction and aims	Method and data	Sampling	Data analysis	Ethics and bias	Results	Transferability or generalizability	Implications and usefulness	Total score	Overall quality
Oostendorp (2017)	4/4	3/3	4/3	3/3	4/3	4/4	4/3	3/3	4/3	33/29	Good
Leighl 2011	4/4	4/4	4/4	4/4	4/4	3/3	4/4	4/4	3/4	34/35	Good
Tang (2008)	3/2	3/3	2/3	3/3	3/3	4/3	2/2	3/3	2/2	25/24	Fair
DuBenske 2010	3/2	4/2	3/2	2/1	2/1	1/1	3/2	3/3	4/4	25/18	Fair
Shirai (2012)	4/4	4/3	3/4	3/4	4/4	4/3	4/4	3/3	3/3	32/32	Good
Smith (2011)	4/4	3/2	3/2	3/3	3/2	4/3	4/3	3/2	3/2	30/23	Fair
Meropol (2013)	3/3	3/4	4/4	4/4	4/4	4/3	3/3	3/4	4/4	32/33	Good

Note

Quality appraisal scores of both researchers are presented with: 1 = very poor, 2 = poor, 3 = fair, 4 = good.
